# A highly efficient ligation-independent cloning system for CRISPR/Cas9 based genome editing in plants

**DOI:** 10.1186/s13007-017-0236-9

**Published:** 2017-10-16

**Authors:** Aftab A. Khan, Ashraf El-Sayed, Asma Akbar, Arianna Mangravita-Novo, Shaheen Bibi, Zunaira Afzal, David J. Norman, Gul Shad Ali

**Affiliations:** 1 Department of Plant Pathology, Mid-Florida Research and Education Center, Institute of Food and Agriculture Sciences, University of Florida, 2725 S. Binion Road, Apopka, FL 32703 USA; 2 Enzymology and Fungal Biotechnology Lab, Faculty of Science, Zagazig University, Zagazig, 44519 Egypt

## Abstract

**Background:**

Most current methods for constructing guide RNAs (gRNA) for the CRISPR/Cas9 genome editing system, depend on traditional cloning using specific type IIS restriction enzymes and DNA ligation. These methods consist of multiple steps of cloning, and are time consuming, resource intensive and not flexible. These issues are particularly exacerbated when multiple guide RNAs need to be assembled in one plasmid such as for multiplexing or for the paired nickases approach. Furthermore, identification of functional gRNA clones usually requires expensive in vitro screening. Addressing these issues will greatly facilitate usage and accessibility of CRISPR/Cas9 genome editing system to resource-limited laboratories.

**Results:**

To improve efficiency of cloning multiple guide RNAs for the CRISPR/Cas9 system, we developed a restriction enzyme- and ligation-independent strategy for cloning gRNAs directly in plant expression vectors in one step. Our method relies on a negative selection marker and seamless cloning for combining multiple gRNAs directly in a plant expression vector in one reaction. In addition, using the *Agrobacterium*-mediated transient assays, this method provides a simple *in planta* procedure for assaying the effectiveness of multiple gRNAs very rapidly.

**Conclusions:**

For a fraction of resources used in the type IIS restriction enzyme-based cloning method and in vitro screening assays, the system reported here allows efficient construction and testing several ready-to-transfect gRNA constructs in < 3 days. In addition, this system is highly versatile and flexible, and by designing only two additional target-specific primers, multiple gRNAs can be easily assembled in any plasmid in a single reaction.

**Electronic supplementary material:**

The online version of this article (doi:10.1186/s13007-017-0236-9) contains supplementary material, which is available to authorized users.

## Background

Precise editing of genomes using sequence-specific nucleases (SSN) has been rapidly adapted by research laboratories throughout the world. This is primarily due to several breakthrough discoveries and developments in DNA editing technologies such as zinc finger nucleases (ZFN) [1, 2], transcription activator-like effector nucleases (TALEN) [3, 4], and clustered, regulatory interspaced, short palindromic repeats (CRISPR) and CRISPR-associated 9 (CRISPR/Cas9) [5–7]. These technologies basically work by first introducing a double stranded DNA break (DSB) in the target site followed by repair of DNA ends by the endogenous DNA break repair mechanisms of non-homologous end joining (NHEJ) or homologous recombination (HR) [8, 9]. NHEJ, which is the most common repair mechanism in eukaryotes, and, which utilizes DNA ligases and other cofactors for joining broken DNA ends, is error-prone often leading to insertion/deletion resulting in genetic mutations in the targeted genomic region [10–12]. HR on the other hand is usually precise and leads to integration of foreign DNA in the target site.

Genome editing tools primarily fall into four major categories namely ZFN [1, 2], TALEN [3, 4], homing endonucleases [13] and CRISPR/Cas9 [6, 7]. ZFNs and TALENs consist of a core nuclease fused in *cis* to a site-specific DNA-binding protein module for directing nuclease to the targeted genomic location. ZFNs and TALENs, which were among the first technologies used for targeted genome editing, are based on artificial bipartite enzyme, which consist of a modular DNA-binding domain and *Fok*I nuclease, in which the DNA binding domain is engineered to recognize specific DNA sequences [1–4, 14–16]. Although, ZFNs and TALENs have been used successfully in various organisms, they involve de-novo engineering of the DNA binding modules for each target gene, which is time consuming and expensive, and require extensive testing [17]. In contrast, CRISPR/Cas9 is a modular system, which similar to ZFNs and TALENs, consists of a core DNA endonuclease, but utilize a *trans*-acting guide RNA (gRNA) for directing nuclease to the intended genome location [18]. gRNA is a chimeric RNA, which consists of a variable 20-nucleotide target-specific protospacer and a non-variable RNA scaffold, which is derived from CRISPR RNA (crRNA) and trans-activating CRISPR RNA (tracrRNA) molecule. This modularity allows easy designing and engineering of gRNA for virtually any locus. The feasibility of recruiting Cas9 to a target site by changing the 20 nucleotides of protospacer, and combining the crRNA with tracrRNA in a chimeric single guide (sgRNA) makes CRISPR/Cas9 an extraordinarily versatile technology for genome editing in microbes, plants and humans [7, 19]. Furthermore, high efficiency of multiplex genome engineering using multiple gRNA sequences targeting different loci simultaneously has also been shown for the CRISPR/Cas9 system [7, 20]. Since, CRISPR/Cas9 is very simple, versatile and almost universally adaptable in all eukaryotes, it has rapidly evolved as a tool of choice for genome editing. It is set to revolutionize medicine, and plant and animal breeding. Basic CRISPR/Cas9-based genome editing systems have been developed in many organisms, and the feasibility and efficiency of CRISPR technology for editing plant genomes have been demonstrated for the model species *Arabidopsis thaliana* [21, 22], *Nicotiana benthamiana* [18, 23], as well as for several crops such as wheat [24], maize [25], rice [26], sorghum [27] and tomato [27].

CRISPR/Cas9 system is part of the natural adaptive immune system in certain bacteria and archaea for protection against invading DNA or phages by cleavage of the foreign DNA in a very precise manner [5, 28, 29]. This acquired immunity is based on integration of short fragments of the invading DNA as spacers between the direct repeats of the CRISPR locus that are transcribed together as CRISPR RNA (crRNA) [30]. The crRNA has an approximately 20-nucleotide complementary region with trans-activating crRNA (tracrRNA) to guide and activate the Cas9 nuclease to cleave the target DNA [31]. For target recognition and cleavage by Cas9, the prerequisite is the presence of protospacer-adjacent motif (PAM) downstream of the protospacer sequence in the target DNA [5, 32]. The sequence of PAM for Cas9 from *S. pyogenes* is generally 5′-NGG-3′ but it is bacterial species-dependent [33, 34].

Currently, most methods for fusing an RNA pol III-controlled promoter and target-specific protospacer in a single gRNA together with the Cas9 gene in one plasmid, depend on traditional cloning using restriction enzymes and DNA ligation. Traditional cloning is time consuming, resource intensive and not flexible. Only specific type IIS restriction enzymes can be used for seamless cloning of protospacer into an gRNA. Type IIS restriction enzymes such as *Bsa*I, for which restriction sites outside the recognition site can be designed such as in the Golden Gate Assembly, alleviate some of these issues [35]. However, it still requires restriction enzyme digestion and DNA ligation steps. These issues are particularly exacerbated when multiple gRNAs have to be assembled in one plasmid such as for multiplexing or for the paired nickases approach, which requires designing two gRNAs. The method reported here and by Vazquez-Vilar et al. [36] resolves some of these issues. Furthermore, identification of positive clones usually requires screening a large number of colonies. Once constructs are made, they cannot be readily moved to a new plasmid with an improved CRISPR/Cas9 or other RNA guided genome-editing tools. RNA guided genome engineering is rapidly evolving and there are ongoing efforts to further improve efficiency of the system and to reduce off-target sites. To alleviate these issues, here we report the development of an improved cloning technique for CRISPR/Cas9 based genome editing. This cloning technique combines the In-Fusion^®^ HD cloning (www.clonetech.com) and negative selection based on the *ccdB* gene [37] for cloning multiple gRNAs directly in a plant expression vector in one reaction. To demonstrate proof-of-concept of our system, we constructed gRNA constructs targeted against an endogenous gene (*NbPDS*, *phytoene desaturase*), a stably integrated transgene (*aquaporin PIP2*-*1*-*mCherry*) and a transiently expressed transgene (*YFP*) in *Nicontiana benthamiana*. In addition, using the *Agrobacterium*-mediated transient expression system in *N. benthamiana*, one can easily and quickly identify a functional gRNA by screening a set of carefully designed gRNAs. This system is also very flexible and can be adapted for virtually any plasmid by redesigning four of the universal primers.

## Results

### Construction of plasmids

**Fig. 1:**
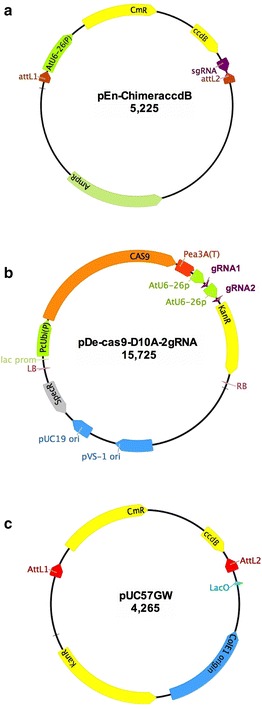
Construction and schematic of plasmid. **a** pEn-Chimera-ccdB. A cassette consisting of chloramphenicol resistance gene (*CmR*) and the *ccdB* gene was PCR-amplified and inserted between the AtU6-26(P) promoter and the sgRNA of pEn-Chimera [22] using the In-Fusion^®^ HD cloning strategy as described in “Methods” section. Plasmid pEn-Chimera-ccdB is used as template in PCR for fusing the 20-nucleotide protospacer sequence to the AtU6-26 promoter and sgRNA. Using the *ccdB* gene virtually eliminated any background colonies, which could arise due to incomplete digestion of pEn-Chimera using the restriction enzymes-based cloning method. **b** pDe-Cas9-D10A-2 gRNA: Schematic illustration of pDe-Cas9-D10A after two gRNA constructs, gRNA1 and gRNA2, are directly cloned in this vector using the In-Fusion^®^ HD cloning system. **c** pUC57GW: this is an in-house constructed Gateway^®^-compatible Entry vector, which, in contrast to commonly used Gateway^®^ Entry/DONR vectors, contains the *ccdB* and Chloranphenicol (*CmR*) resistance genes. This unique design allows efficient cloning of gRNAs constructs in this vector using the In-Fusion^®^ HD cloning system without any background colonies. Please see “Methods” and “Results” section for details

In order to develop an efficient system for cloning gRNA directly into a plant expression vector, we utilized the negative selection marker gene *ccdB* and the In-Fusion^®^ HD cloning strategy. When used as template for cloning an gRNA using type IIS restriction enzymes, the undigested template plasmids, in our case pEn-chimera [22], is also transformed into *E. coli* resulting in a larger number of background colonies. This requires screening large number of colonies for identifying the correct clones containing the gRNA protospacer. In this report, we developed an In-Fusion^®^ HD enzyme-based method, which does not require restriction enzyme digestion of plasmids. For this, we first modified the pEn-Chimera vector by cloning the *ccdB* and chloramphenicol resistance (*CmR*) genes between the AtU6-26 promoter and the scaffold of sgRNA (Fig. 1). The correct orientation and sequence of *ccdB* and *CmR* genes in this plasmid were verified by sequencing. This plasmid, which is named as pEn-Chimera-ccdB, can be maintained in any ccdB resistant *E. coli* strain. It was used as a template for fusing the 20-nucleotide protospacer with the AtU6-26(P) promoter and the scaffold of the sgRNA using high-fidelity PCR and the In-Fusion^®^ HD cloning system as described in the following sections. This plasmid, when transformed into non-ccdB resistant *E. coli* cells, such as the Stellar^®^ cells, did not allow development of any colonies, thus verifying that the *ccdB* gene is functional and that any carried over template plasmid during the In-Fusion^®^ HD cloning will not result in background colonies; see “Methods” section for details. Since we used PCR for amplifying sgRNA and AtU6-26 promoter using pEn-Chimera-ccdB as template, the *ccdB* gene helped eliminate the background colonies in In-Fusion^®^ HD cloning.

Although, the system developed here is robust for direct cloning gRNAs in a plant expression vector, we also developed a unique Gateway^®^-compatible entry vector, pUC57GW, for use with the Gateway^®^ LR cloning system. The pUC57GW plasmid contains the *ccdB* and *CmR* genes between the attL1 and attL2 sites (Fig. 1). This vector was specifically designed and constructed for streamlining cloning a gene of interest and gRNAs in a Gateway^®^-compatible entry vector using In-Fusion^®^ HD cloning as described below.

### Cloning one gRNA for use with Cas9

**Fig. 2:**
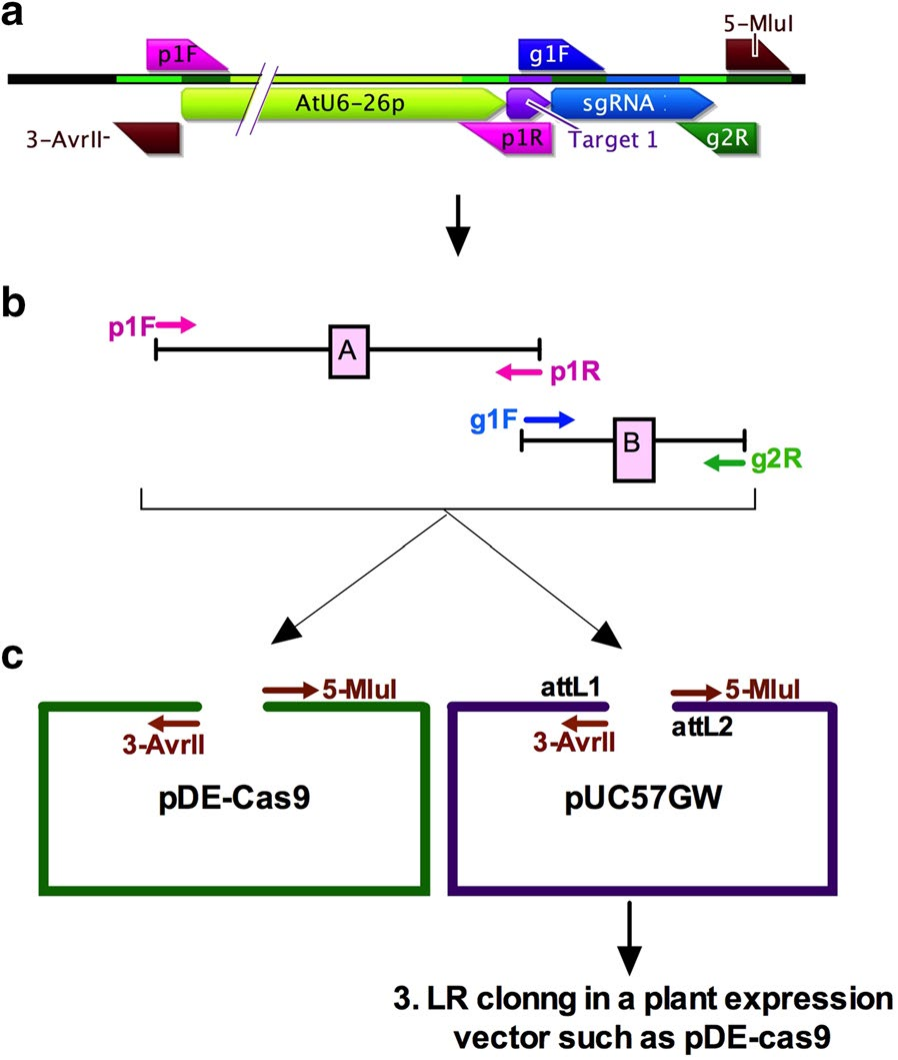
Overview of In-Fusion enzyme-based cloning of a single sgRNA. **a** Schematics of the final assembled CRISPR guide RNA cassette, along with the location of primers is shown in the top panel. **b** Using a set of two universal primers (p1F and g2R) and two sgRNA-specific primers (g1F and p1R), two fragments, A and B, are PCR-amplified using pEN-Chimera-ccdB plasmid as a template that contains AtU6-26(P) promoter and gRNA separated by the *ccdB* gene (Fig. 1). This PCR incorporates the 20-nt protospacer sgRNA sequence to the 3′ end of AtU6-26 promoter in fragments A, and a 15-bp overlap of the 3′ end of the protospacer sgRNA to the 5′ end of fragment B. **c** These two fragments are then fused using the In-Fusion^®^ HD cloning kit with the Cas9-containig pDe-Cas9 fragment, which is amplified with primers 3-AvrII and 5-MluI or obtained by digestion with *Avr*II/*Mlu*I restriction enzymes. Alternatively, fragments A and B can also be fused with pUC57GW amplified with primers 3-AvrII and 5-MluI, which contains the attL1 and attL2 sites for subsequent Gateway^®^ LR cloning in a plant expression destination vector that contains R1 and R2 sites such as pDe-Cas9

Mutating a genomic site requires transcription of one gRNA, which guides Cas9 to the target site. Transcription of chimeric gRNA is usually accomplished by RNA pol III promoters, which control transcription of small non-coding RNAs. In plants, the 20-nucleotide target-specific protospacer sequence is usually cloned behind the AtU6-26 promoter using type IIS restriction enzymes and DNA ligation. This strategy requires more time and could be improved. Instead, we devised a highly efficient, robust and a low-cost cloning strategy, which utilizes the ligation-independent In-Fusion^®^ HD cloning enzyme. This strategy is illustrated in Fig. 2, and it requires only two target-specific primers, p1R and g1F, and a set of two universal primers, p1F and g1R (Table 1). The primers are designed in such a way that each of the two overlapping primers, which are located at the ends of to-be-fused two adjacent fragments, includes a unique 15-bp overlap sequence at the 5′ end, which is required for In-Fusion^®^ HD cloning. The backbone of the gRNA plasmid, such as pDe-Cas9 or pUC57GW (Fig. 1), which carry a 15-bp overlap at the 5′ end (compatible with the 3′ end of the sgRNA cassette), and a 15-bp overlap at the 3′ end (compatible with the 5′ end of the AtU6-26 promoter), can be either PCR-amplified using two universal primers (3-AvrII and 5-MluI, Table 1) or can be obtained by digestion with *Mlu*I and *Avr*II enzymes. We have successfully used both methods for making gRNA constructs targeted against the *Yellow Fluorescent Protein (YFP)* gene. We were also successful in scaling down the In-Fusion^®^ HD cloning reactions by at least 6 times. Scaling down not only reduced costs but also eliminated several reaction dilution steps in the In-Fusion^®^ HD cloning protocol. Using only 1/6th of the recommended reagents in the In-Fusion^®^ HD cloning kit, we obtained more than 100 colonies for each construct in these vectors. Restriction digestion of plasmids isolated from these colonies revealed the expected RFLP patterns in 100% (4 of 4) plasmids. Sequencing of the resulting binary vector, pDE-Cas9-gYFP1, and of the Gateway^®^-compatible entry vector pUC57GW-gYFP1 verified the correct insertion of the protospacer with the scaffold of sgRNA behind the AtU6-26 promoter in both plasmids. In conclusion, using In-Fusion^®^ HD cloning system in conjunction with only three PCR reactions, two for constructing the gRNA fragments, and one for amplifying the destination vector, one can easily assemble several ready-to-transfect constructs in < 3 days.

**Table 1:**
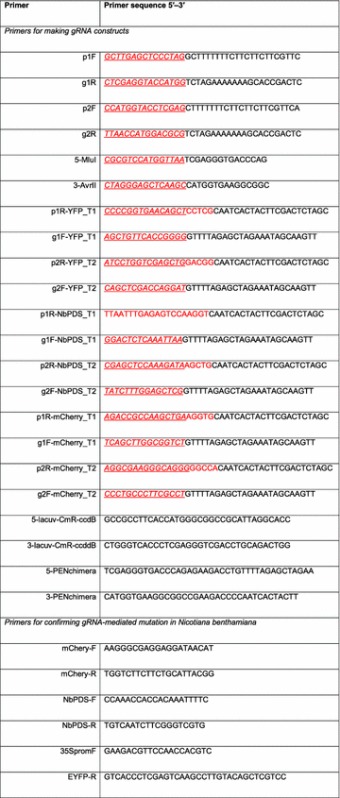
List of primers used in this study

The 15-bp overlaps, required for In-Fusion® HD cloning, are shown in italic and underlined, and gene-specific protospacer for Target 1 and Target 2 are shown in red font

### Cloning two gRNAs for use with the paired Cas9-D10A nickase or for multiplexing

**Fig. 3:**
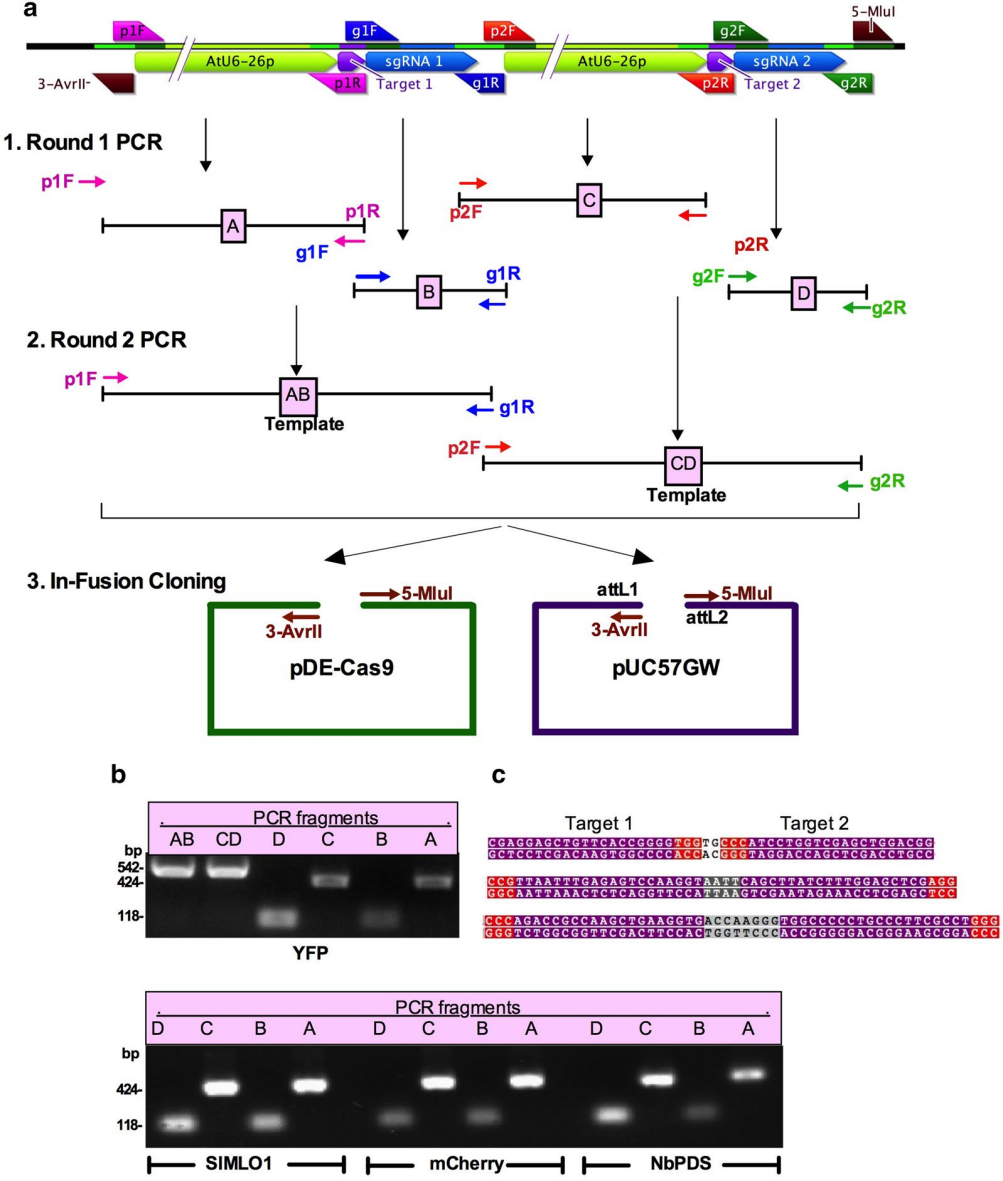
Overview of In-Fusion^®^-based cloning of two gRNA targets for paired nickases (Cas9-D10A). **a** Illustration of cloning strategy. Schematics of final gRNA cassette is shown in the top panel. Using a set of four universal primers (p1F, p2F, g1R and g2R) and four target-specific primers (g1F and p1R for protospacer target 1, and g2F and p2R for protospacer target 2), four fragments, A, B, C and D are PCR amplified using pEn-Chimera-ccdB plasmid in Round 1 PCR. In Round 2 PCR, using primers p1F and g1R, fragments A and B are fused resulting in fragment AB, and using primers p2F and g2R, fragments C and D are fused resulting in fragment CD. In Step 3, fragments AB and CD are cloned into pDe-Cas9-D10A or pUC57GW using the In-Fusion^®^ HD cloning system. **b** A representative gel picture showing PCR fragments of *YFP* upper panel, SlMLO1, *NbPDS* and *mCherry* lower panel. Expected sizes of each fragment are shown on the left. **c** Protospacer sequences of the targeted genes (*YFP* upper panel, *NbPDS* middle panel, and *mCherry* lower panel) are highlighted in purple background and the PAM sequences NGG in red background

The 20-nucleotide target-specific protospacer sequence of a single gRNA might occur randomly in eukaryotic genomes, which could result in mutating unintended off-targets. To reduce mutations in off-target sites, a strategy, which utilizes dual-RNA-guided paired nickases, has been reported [5, 19, 38–40]. This strategy utilizes a mutated version of Cas9, which carries a point mutation (D10A) in the RuvC-like domain, and which, in contrast to Cas9, produces nicks in only one DNA strand [5, 38]. By targeting a pair of Cas9-D10A nickases to two different adjacent sites on opposite DNA strands of a genomic locus creates long single-stranded overhangs, essentially producing a staggered double stranded break (DSB), which is then repaired by the endogenous DNA break repair mechanisms. This strategy requires designing and constructing two gRNAs, which target two closely located genomic regions. In most current published methods, the two gRNAs are cloned sequentially in a plant expression vector using a combination of restriction enzyme-based cloning for one gRNA and the Gateway^®^ cloning strategy for the other gRNA [25]. In another method, the individual gRNAs are first cloned in individual vectors, which are then combined using the Golden Gate cloning strategy [20]. Both these strategies are efficient and simple but still need improvements. Utilizing the In-Fusion^®^ HD cloning strategy, we devised an extremely simple and robust method that allows cloning two gRNAs in one simple reaction directly in the pDe-Cas9-D10A or pDe-Cas9 vector, which contains the Cas9 cassette [22]. Basic step in this strategy involves designing a set of four target-specific and four universal primers. Overall cloning strategy for cloning two gRNAs in the same vector is illustrated in Fig. 3. A generalized design and locations of primers in relation to the final gRNA construct are illustrated in Additional file 1: Figure S1.

We conducted proof-of-concept experiments for the paired nickase strategy targeting an endogenous *N. benthamiana* gene (*NbPDS*, *phytoene desaturase*), a transgene (a membrane localized chimeric “*aquaporin PIP2*-*1*-*mCherry*” gene) stably expressed in *N. benthamiana*, and a transiently expressed gene (*YFP*) carried on a plant expression vector (pGWB415-HA-YFP). For each of these three genes, we chose a pair of 20-nucleotide protospacer sequences targeting two regions, Target 1 and Target 2 on the opposite strands of the target genes (Fig. 3c). Both Target 1 and Target 2 are followed by protospacer adjacent motif (PAM) sequence NGG in the target gene. An important step in designing the CRISPR/Cas9-based constructs is choosing appropriate target sites. A number of online tools are available for designing gRNA [41], and we chose to use a free-of-cost gRNA design tool available online (DNA 2.0 Inc, https://www.dna20.com/). The 20 nucleotide protospacer was fused to the scaffold of sgRNA and the *A. thaliana* U6-26 promoter [38] using high-fidelity PCR and In-Fusion^®^ HD cloning strategy as follows. Using appropriate primer pairs (listed in Table 1 and Fig. 3) and pEn-Chimera-ccdB as template, we PCR amplified four fragments, labeled as A (5′-AtU6-26p-sgRNA1-3′), B (5′-sgRNA1-3′), C (5′-AtU6-26p-sgRNA2-3′) and D (5′-sgRNA2-3′) in Fig. 3a (Round 1 PCR). In a second round of PCR, we fused fragments A and B into one fragment, AB, using fragments A and B as templates with primers p1F and g1R according to the strategy illustrated in Fig. 3a. Similarly, fragments C and D were fused together into fragment CD using fragments C and D as templates with primers p2F and g2R. Backbone of the gRNA vector pDe-Cas9-D10A was either PCR amplified using primers 5-MluI and 3-AvrII or obtained by digestion with restriction enzymes *Avr*II and *Mlu*I. Fragments AB, CD and the pDE-Cas9-D10A backbone were fused together using the In-Fusion^®^ HD cloning kit.

The In-Fusion^®^ HD cloning method can be performed with either unpurified or gel-purified fragments. In preliminary experiments, using unpurified fragments yielded an average 4.5 colonies in three independent reactions. Restriction fragment length polymorphism (RFLP) analyses of plasmids from 8 randomly picked colonies revealed 2 colonies with the expected RFLP patterns. In contrast, with gel-purified fragments we consistently obtained more than 200 clones in one single reaction. Plasmids from five independent colonies were digested with *Not*I/*Mlu*I. As shown in Additional file 1: Figure S2 (lanes B1–B5), the expected RFLP pattern consisting of 9691, 3219, 1536 and 1294 bp fragments were observed with all five plasmids reflecting a 100% success rate. DNA sequencing of these plasmids verified correct orientation and sequence of all components of gRNAs, including the AtU6-26 promoters, the protospacer and scaffold of sgRNAs in pDE-Cas9-D10A. In conclusion, using this strategy, screening only one or two clones are needed for identifying correct gRNA constructs further reducing costs and time.

### Optimization of molar ratios of fragments to vectors

Experiments for finding optimal molar ratios of the fragments relative to vector revealed high success rates (more than 50 colonies per reaction) with a wide range of molar ratios of fragments (15–180 fmoles) and vector (15–90 fmoles) per 1.8 µl reaction volume. This allows very little adjustment of concentrations of fragments and vector before the In-Fusion^®^ HD reaction is performed. High success rate with a wide range of molar ratio is likely due to high efficiency and specificity of the In-Fusion^®^ HD cloning system. Since using different vector to fragment molar ratios did not reveal significant difference in cloning efficiency, in subsequent experiments we proceeded with the In-Fusion^®^ HD cloning without adjusting molar ratios of individual fragments.

Taking advantage of the observation that a wide range of molar ratios of fragments can be used, we also tested to directly clone all four fragments A, B, C and D without going through the Round 2 PCR in a single In-Fusion^®^ HD reaction. For this, all four fragments were gel-purified together on a single column of QIAquick^®^ Gel Extraction Kit (cat. nos. 28704). RFLP analyses of gRNA plasmids resulting from this strategy using *Not*I/*Mlu*I restriction enzymes, revealed that 3 of 5 clones were accurate reflecting a 60% success rate. Thus skipping the Round 2 PCR still yields positive clones but at a lower success rate.

### Cloning gRNAs by combining Gateway^®^ and In-Fusion^®^ HD cloning

Since genome editing systems are rapidly evolving with continuous improvements, we also devised a strategy for cloning gRNA constructs in a Gateway^®^-compatible entry vector for subsequent subcloning in a Gateway^®^-compatible destination plasmid. For this, we utilized the same primers as those designed for direct cloning in pDe-Cas9-D10A vector. The In-Fusion^®^ HD cloning strategy is basically the same as for pDe-Cas9-D10A except that we used a PCR-amplified backbone vector pUC57GW that carries the attL1 and attL2 sites for the downstream Gateway^®^ LR cloning reaction (Fig. 3). pUC57GW, in addition to attL1 and attL2 sites, also carries the *ccdB* gene for improving efficiency of obtaining only positive clones. The *ccdB* gene, which is excluded from the backbone vector after PCR amplification using primers 5-MluI and 3-AvrII (Table 1), allows reducing background colonies. To confirm positive clones, we digested plasmids from 5 single colonies with *Not*I/*Mlu*I restriction enzymes. As is shown in Additional file 1: Figure S2 (lanes A1-A5), all clones displayed the expected RFLP patterns consisting of 2811 and 1046 bp bands. Two clones were also verified by sequencing, which revealed correct orientation of all DNA sequences including the AtU6-26 promoters, Target 1 and Target 2, and the scaffold of sgRNAs. Using the Gateway^®^ LR reaction, this construct was moved to the Gateway^®^-compatible destination vector pDe-Cas-D10A vector. Overall, these results suggest that the multiple gRNA can be quickly assembled in Gateway^®^- Entry vector, from where they can then be transferred into any desired Gateway^®^-compatible destination vector.

### In vivo verification of the gRNA constructs using the *Agrobacterium*-mediated transient system in *N. benthamiana*

**Fig. 4:**
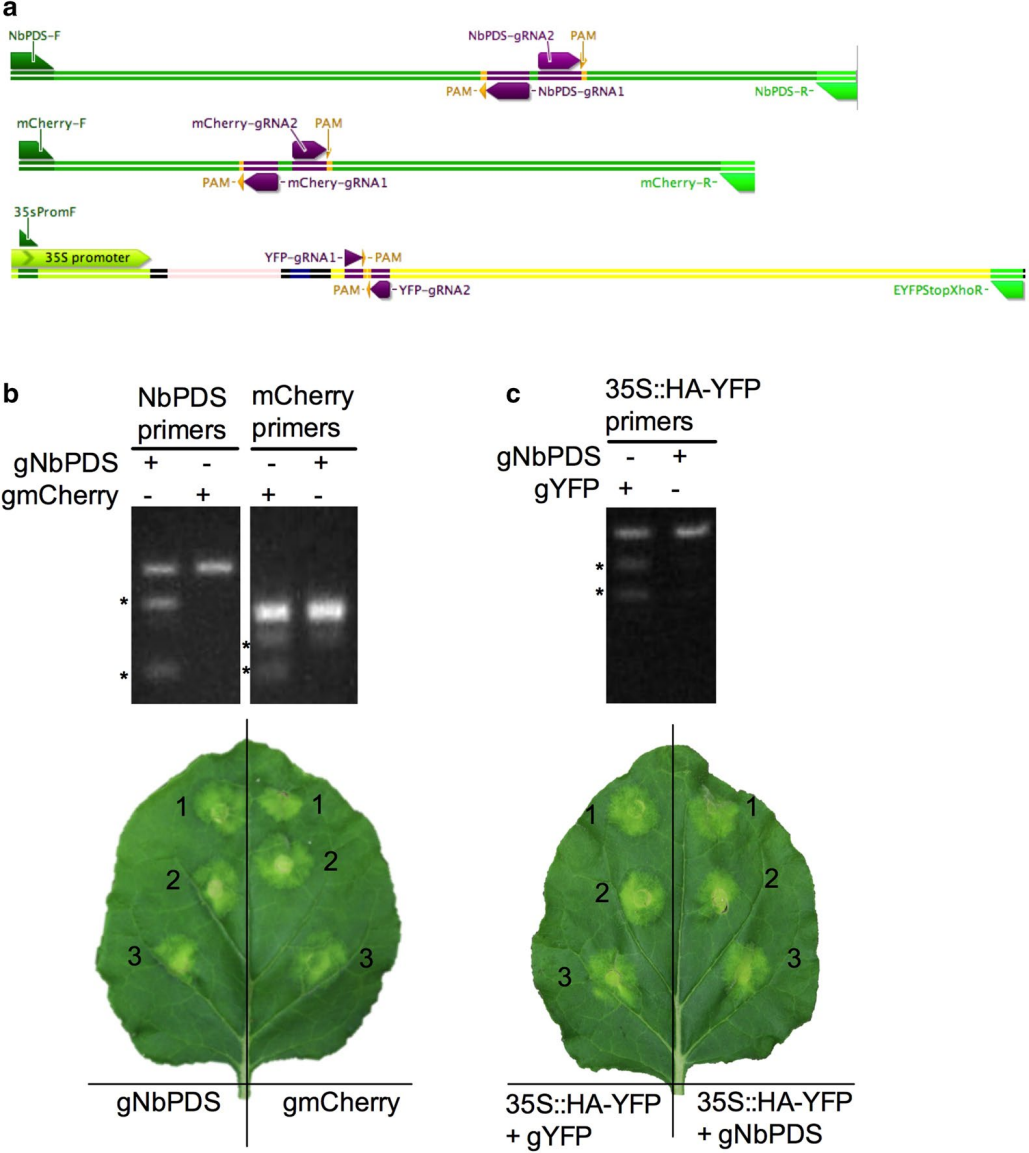
Mutation detection using *Agrobacterium*-mediated transient expression in *N. benthamiana.* **a** Schematic illustrations showing the locations of gRNAs, PAM positions, and primers in the genes*, NbPDS*, *PIP2*-*1*-*mCherry* and *YFP* targeted for genome editing. **b** Resolvase assay for detecting mutation in *NbPDS* and *mCherry* targeted by the gRNA constructs pDe-Cas9-D10A-gNbPDS and pDe-Cas9-D10A-gmCherry. High fidelity PCR was performed with primers NbPDS-F and NbPDS-R for the NbPDS target, and mCherry-F and mCherry-R for the mCherry target (panel A and Table 1) using template DNA isolated from leaf spots on *N. benthamiana* infiltrated with *Agrobacterium tumefaciens* carrying the indicated CRISPR–Cas9 constructs; *N. benthamiana* was stably transformed with the *PIP2*-*1*-*mCherry* gene, which served as a transgene target for the gmCherry gRNA. Amplicons were subjected to Resolvase assays (Guide-it™ Mutation Detection Kit, Cat#631443, Clontech) and reactions were run on a 1.5% agarose gels. Digested fragments, which result from gRNA-induced mutations, are indicated by *, and their sizes are ~ 570 and ~ 150 bp for NbPDS, and ~ 277 and ~ 150 bp for mCherry. Undigested fragments are 727 bp for NbPDS and 427 bp for mCherry. Lower panel shows a representative *N. benthamiana* leaf infiltrated in three independent sites with the indicated CRISPR–Cas9 constructs. **c** Surveyor (CEL II)—nuclease assay for detecting mutation in a transiently transformed *YFP* gene carried on pGWB415-35S::HA-YFP plasmid. PCR was performed with forward primer (35SpromF) and reverse primer (EYFPStopXhoR) (panel A and Table 1) using template DNA from Wild Type *N. benthamiana* co-transformed with pGWB415-35S::HA-YFP along with either pDe-Cas9-D10A-gYFP or pDe-Cas9-D10A-gNbPDS (negative control). Surveyor (CEL II)—nuclease assay was performed with amplicons and reactions were run on 1.5% agarose gels (mismatch-specific Surveyor nuclease, Surveyor^®^ Mutation Detection Kit; Cat#706025, IDTdna.com). gRNA-induced mutations are revealed by the digested ~ 700 and ~ 400 bp fragments, marked by *; the undigested fragment is 1067 bp, which is shown in both lanes. Lower panel shows a representative *N. benthamiana* leaf infiltrated in three independent sites with the indicated CRISPR–Cas9 constructs

The functionality of a specific gRNA can be tested in vitro using purified Cas9 enzyme and a DNA fragment containing the target sequence. However, in vitro results may not necessarily hold true in living cells. In this report, we developed an easy-to-use in vivo method for testing the functionality of designed gRNA constructs using the *Agrobacterium*-mediated transient expression system in *N. benthamiana*. The gRNA constructs, pDe-Cas9-D10A-gNbPDS and pDe-Cas9-D10A-gmCherry were separately infiltrated into *N. benthamiana* stably expressing a chimeric aquaporin *PIP2*-*1*-*mCherry* gene [42, 43]. If the NbPDS gRNA is functional it should mutate the endogenous *NbPDS* gene in some cells in the infiltrated areas. Similarly, the mCherry gRNA should mutate the *mCherry* gene. These hypotheses were tested by conducting resolvase nuclease assays with DNA isolated from the *Agrobacterium*-infiltrated leaf spots. As expected, the non-mutated 727 bp fragment was PCR-amplified from leaf spots infiltrated with either pDe-Cas9-D10A-gNbPDS or pDe-Cas9-D10A-gmCherry (Fig. 4c). The expected ~ 570 and ~ 150 bp fragments, which result due to gNbPDS-induced mutations, were observed only in the leaf spots infiltrated with pDe-Cas9-D10A-gNbPDS. Similarly, the expected 427 bp non-mutated fragment for the mCherry transgene was PCR-amplified from both the pDe-Cas9-D10A-gNbPDS and pDe-Cas9-D10A-gmCherry-infiltrated leaf spots, but the expected gmCherry-induced ~ 277 and ~ 150 bp fragments were observed only in the pDe-Cas9-D10A-gmCherry-infiltrated leaf spots.

We also explored the possibility of testing the mutation of an exogenous gene (*YFP*) carried on a binary plasmid when transiently transferred into *N. benthamiana*. As is shown in Fig. 4c, co-expression of pDe-Cas9-D10A-gYFP along with its target *YFP* gene (carried on pGWB415-HA-YFP) resulted in the expected gYFP-induced ~ 700 and ~ 400 bp fragments, along with the non-mutated 1067 bp fragment. In control leaf spots, infiltrated with pDe-Cas9-D10A-sgPDS along with the unintended *YFP* gene (carried on pGWB-HA-YFP) no mutations were detected. These results show that the functionality of gRNA constructs made using the In-Fusion^®^ HD cloning strategy can be quickly assayed using the *Agrobacterium*-mediated transient expression system in *N. benthamiana*.

## Discussion

The CRISPR/Cas9 system has rapidly become a method of choice for genome editing in basic and applied research in various organisms. The type II CRISPR/Cas9 system consists of a fixed core RNA-guided DNA nuclease, Cas9, and a chimeric gRNA consisting of a non-variable RNA scaffold derived from the crRNA and tracrRNA and a target-specific 20-nucleotide protospacer RNA, which is engineered specifically for a genomic region to be edited. The protospacer for each target has to be cloned between an RNA pol III-controlled promoter and the scaffold of sgRNA. The gRNA construct is then usually cloned in a plant expression vector, which carries the Cas9 gene on the same T-DNA cassette. Current methods for cloning the target-specific protospacer sequence with an RNA pol III-controlled promoter and the scaffold of sgRNA are time consuming and involve multiple expensive cloning steps utilizing type IIS restriction enzymes [38, 40]. Ligation-dependent methods are also prone to getting a larger number of background colonies resulting from the undigested parent vector. This requires screening many colonies to identify positive clones consuming resources and time. The system developed here utilizes negative selection based on the *ccdB* gene. The *ccdB* gene codes for a toxin, which is part of toxin-antitoxin system that inactivates DNA-topoisomerase II complexes thus killing host *E. coli* cells that do not carry the antitoxin *ccdA* gene [44]. Taking advantage of this property, multiple cloning methods such as the Gateway^®^ cloning methods have been developed for reducing background colonies. We developed a unique template plasmid, pEN-Chimera-ccdB carrying the *ccdB* gene, which virtually eliminated any background plasmids thus greatly accelerating the cloning process for making multiple gRNAs. Using this plasmid as a template, we have developed a robust cloning method for direct cloning of gRNAs in a plant expression vector using the ligation independent In-Fusion^®^ cloning system. The In-Fusion^®^ enzyme uses a 15-bp overlap between to-be-fused fragments, which can be easily incorporated into primers that are used for PCR amplification [45, 46]. Although, the In-Fusion^®^ cloning method can be used for fusing several fragments in one reaction [46], in our experience, increasing the number of fragments leads to reduced number of positive clones. This issue was easily resolved conducting an additional round of overlapping PCR to fuse adjacent fragments in a sequential manner. The additional round of PCR utilizes the same primers that are used for amplifying the component fragments thus eliminating the need for designing any new primers. The gRNA cloning system developed here greatly expedites cloning multiple gRNAs in any Cas9-containing vector directly. Compared to the time and resources used in the type IIS restriction enzyme-based cloning, this method will greatly expedite the cloning of multiple gRNA constructs.

The plasmids that we developed are for use in plants but the cloning technique can be adapted for any vector system in any other organism redesigning only four universal primers. The gRNA cloning strategy, which we demonstrated for targeting two sites, can be easily expanded for multiplexing where simultaneous introduction of multiple gRNAs for targeting multiple loci is desired. Currently, multiple gRNAs are constructed using the Golden Gate cloning technology or other systems, but they still involves multiple steps of digestion and ligation prolonging and complicating the cloning processes [47]. Similarly, other systems such as separating multiple gRNAs by tRNAs driven by the same promoter has also been reported [48]. However, this system also needs multiple steps of cloning. In contrast, using our system an additional gRNA expression cassette can be easily added by designing four additional primers. This process can be repeated multiple times for adding additional gRNAs in the same construct.

Testing the functionality of gRNAs before they are stably transformed into plants is essential. In this report, we showed that the functionality of gRNAs can be easily tested in a simple *Agrobacterium*-mediated transient expression in *N. benthamiana*. This system is superior to the currently used in vitro methods for assaying the functionality of gRNA in several ways. First, using an in vivo system, we obtained direct evidence for the efficiency of an gRNA in mutating the intended target locus in the targeted plant species. Second, in contrast to the in vitro gRNA mutation detection systems, which require purified Cas9 enzyme and the intended target gene template, the in vivo system reported here expresses the Cas9 enzyme encoded by the *Cas9* gene and the target gene is either an endogenous gene or it can be easily co-transferred along with the Cas9 gene on another plant expression vector such as we demonstrated for the YFP gene. This allows rapidly screening multiple gRNAs with less resources.

New developments are regularly being made in discovering newer CRISPR-Cas systems [49–51], which are expected to improve the applications of the CRISPR system in diverse biological disciplines. Similarly, in addition to genome editing, the Cas9 system has also been used for other applications such as transcriptional control [52, 53] and visualization of genomic loci [54, 55] and epigenome editing [56] by using nuclease-inactive deactivated dCas9. These findings will inevitably lead to the development of new vector systems or modification of existing vectors such as by replacing the existing Cas9 with improved versions. The versatility and flexibility of our system would easily allow adaptations to any exiting or future vectors containing the desired Cas9 nucleases.

## Conclusions

The method developed here, provides a simple and robust procedure for cloning multiple guide RNAs for use with CRISPR/Cas9-based genome-editing in one simple reaction using the In-Fusion^®^ HD cloning system. In contrast to currently used methods, our method does not require any restriction enzymes or ligation reactions. The method developed in this manuscript is highly flexible and versatile and allows assembling and testing multiple sgRNAs very rapidly in vivo in any vector thus greatly reducing the time and resources utilized in the currently used methods.

## Methods

### Construction of plasmid pEn-Chimera-ccdB

Plasmid pEn-Chimera [22] was used as a backbone for constructing pEn-Chimera-ccdB. The ccdB and chloramphenicol resistance (CmR) genes were cloned between the AtU6-26 promoter and the scaffold of gRNA in plasmid pEn-Chimera using the In-Fusion^®^ HD^®^ HD Cloning Kit (Clontech) as follows. First, a fragment encompassing genes *CmR* and *ccdB* was amplified from a Gateway^®^-compatible destination vector (pGSA002-nYFPn) using CloneAmp™ 2 × HiFi PCR mix (Cat# 639298, Clontech) according to the manufacturer protocol with slight modifications as follows. Each PCR reaction consisted of a 10 µl total volume, and contained 240 nM Forward primer (5-lacuv-CMR-ccdB, 2 µl of a 1.2 µM stock) and 240 nM Reverse primer (3-lacuv-CMR-ccdB, 2 µl of a 1.2 µM stock), 100 pg template plasmid (pGSA002-nYFPn, 1 µl of 0.1 ng/µl stock), and 5 µl of 2 × CloneAmp™ HiFi PCR Premix. Primer sequences are listed in Table 1. PCR reactions were performed at 98 °C for 10 s, 55 °C for 15 s and 72 °C for 1.5 min for 35 cycles. Similarly, the plasmid pEn-Chimera backbone was amplified using 240 nM of primers 5-PENchimera and 3-PENchimera (each 2 µl of a 1.2 µM stock, Table 1), 20 pg plasmid pEN-Chimera (1 µl 20 pg/µl) and 5 µl 2 × CloneAmp™ HiFi PCR Premix using PCR conditions as above. Expected fragments (*ccdB* and *CmR* genes, 1569 bp and pEN-Chimera backbone, 3738 bp) were gel purified (QIAquick Gel Extraction Kit, Cat#28706, Qiagen) and cloned together using the In-Fusion^®^ HD cloning Kit (Cat#639648, Clontech) according to manufacturer’s instructions. Following confirmation with RFLP analysis, plasmids were verified by DNA sequencing. This vector was used as a template for making gRNA constructs.

### Designing and cloning gRNA in pUC57GW, pDe-Cas9 and pDe-Cas9-D10A

To provide a proof-of-concept for our strategy, we designed one and two gRNAs targeted against a transiently expressed gene (*YFP*), an endogenous gene (*NbPDS*) and a stably integrated transgene (*PIP2*-*1*-*mCherry*) in *N. benthamiana*. For the Cas9-D10A nickase, two 20 nucleotides-long guide RNAs (gRNA) for each gene (YFP, NbPDS and mCherry) were selected using an online CRISPR gRNA designing tool (DNA 2.0 Incorporation; https://www.dna20.com/eCommerce/Cas9/input). Both target sequences along with the PAM sequences are highlighted in Fig. 3c. A generic In-Fusion^®^ HD cloning strategy of cloning single gRNA (gRNA 1) for Cas9, or two gRNAs, gRNA1 and gRNA2 for Cas9-D10A are shown in Figs. 2 and 3, respectively. The In-Fusion^®^ HD cloning system fuses any DNA fragments with 15-bp overlaps at their ends. The 15-bp overlap in the vector can be generated either by PCR or using restriction enzymes. The pDe-Cas9-D10A was digested with *Avr*II and *Mlu*I resulting in a 14713 bp linear fragment with different 15-bp overlaps at the 3′ and 5′ ends for fusing with the YFP, PDS and mCherry gRNAs. The *Avr*II/*Mlu*I digested pDe-Cas9-D10A fragment was gel-purified (QIAquick Gel Extraction Kit, cat:28706, Qiagen) and stored at − 20 °C until further use. One or two rounds of high fidelity PCR reactions were then performed for fusing the gRNAs with the *A. thaliana* AtU6-26 promoter. Using pEn-Chimera-ccdB plasmid as template, two fragments (A and B, Fig. 2), or four fragments (A, B, C and D, Fig. 3) were amplified in separate PCR reactions using the CloneAmp 2 × HiFi PCR mix (Clontech) and PCR conditions as described above. Each reaction consisted of pEn-Chimera-ccdB (1 µl of 20 pg/µl) as template, 240 nM Forward and Reverse primers (each at 2 µl of a 1.2 µM stock) and 5.0 µl of 2 × CloneAmp™ 2 × HiFi PCR premix. All fragments contained 15-bp overlaps at their ends in a way that resulted in directional fusion of fragment 5′-A-B-3′ or 5′A-B-C-D-3′. Primer p1F and p1R amplified a 427 bp fragment A with a 5′ 15 bp overlap with the 3′ end of pDe-Cas9, pDe-Cas9-D10A or pUC57GW, and a 3′ 15-bp overlap with the 5′ end of fragment B. Primers g1F and g1R amplified a 118 bp fragment B with a 5′ 15 bp overlap with 3′ end of fragment A, and a 3′ 15 bp overlap with the 5′ end of fragment C. Primers p2F and p2R amplified fragment C with a 5′ 15 bp overlap with the 3′ end of fragment B, and a 3′ 15 bp overlap with 5′ end of fragment D. Primer g2F and g2R amplified a 118 bp fragment D with a 5′ 15 bp overlap with fragment C and a 3′ 15 bp overlap with the 5′ end of pDe-Cas9, pDe-Cas9-D10A or pUC57GW. All four fragments were diluted to 1/20th with ddH_2_O and used as templates in a second of round of overlap PCR for fusing fragments A and B to produce fragment AB consisting of 5′-AtU6-26p-gRNA1-3′ using primers p1F and g1R, and fragments C and D to produce fragment CD consisting of 5′-AtU6-26p-gRNA2-3′ using primers p2F and g2R. Both fragments AB and CD were either used directly or gel purified in the In-Fusion^®^ HD reaction for cloning in pDe-Cas9-D10A or a Gateway^®^ compatible entry vector, pUC57GW. For cloning in pUC57GW, the vector backbone was amplified using forward primer 5-MluI and reverse primer 3-AvrII. This amplifies a 2838 bp linear vector fragment, which contains 5′ and 3′ 15-bp overlaps with the 3′ and 5′ ends of fragment A and D, respectively. The vector backbone fragment was used either directly or gel purified for use in the In-Fusion^®^ HD reaction for fusing fragments AB and CD. Since products AB and CD have 15-bp overlaps with each other and with the vector at two ends, they can be cloned in vector pUC57GW using the In-Fusion^®^ HD reaction. Infusion reactions were assembled according to the manufacturer’s protocols with slight modifications for scaling down reaction volumes as follows. Each In-Fusion^®^ HD reaction consisted of 0.5 µl fragment AB (15–180 fmoles), 0.5 µl fragment CD (15–180 fmoles), 0.5 µl pUC57GW vector backbone (15–90 fmoles), and 0.3 µl of 5 × infusion HD enzyme mix. In-Fusion reaction was performed at 50 °C for 15 min and inactivated at 80 °C for 10 min. One µl of the In-Fusion reaction was transformed into 20 µl Stellar™ (Cat#676367, Clontech) competent cells and plated on LB plates containing 50 µg/ml kanamycin. Five independent colonies were selected and plasmids were isolated using QIAprep Spin Miniprep kit (Cat#27105). Plasmids were digested with appropriate restriction enzymes for RFLP analyses for identifying positive clones. For cloning in plasmid pDe-Cas9-D10A, we used two strategies. In one strategy, we used the Round 2 PCR products AB and CD along with *Avr*II/*Mlu*I digested pDe-Cas9-D10A fragment. The infusion reaction setup and transformation into Stellar™ cell was exactly as described above for cloning in pUC57GW. In the second strategy, the Round 1 four PCR products A, B, C and D were run on a gel, and all bands mixed together in one tube and gel-purified using the QIAgen Gel Purification kit. These gel-purified products were then used in In-Fusion^®^ HD reaction consisting of 1 µl of combined products A, B, C and D (~ 30 fmoles), 0.5 µl of digested pDe-Cas9-D10A (30 fmoles), and 0.3 µl of 5 × In-Fusion^®^ HD enzyme mix. In-Fusion^®^ HD reaction and transformation into Stellar™ competent cells were performed exactly as described above. Five to eight single clones using each strategy were digested with enzymes *Mlu*I and *Not*I for RFLP analyses. Putative positive clones were verified by sequencing on both strands.

### In vivo verification of induced mutation using *Agrobacterium*-mediated transient expression in *N. benthamiana*

pDe-Cas9-D10A-gYFP, pDe-Cas9-D10A-gNbPDS, and pDe-Cas9-D10A-gmCherry were transformed separately into *Agrobacterium tumefaciens* GV3101. *A. tumefaciens* strains carrying pDe-Cas9-D10A-gNbPDS or pDe-Cas9-D10A-gmCherry were adjusted to OD_600_ = 1.0, and infiltrated with a needless syringe into fully expanded leaves of 4-week old *N. benthamiana* transgenic plants expressing a PIP2-1-mCherry transgene. 10 days later DNA was extracted from the infiltrated area, which was tested for the intended mutation using a mismatch-specific Surveyor nuclease (Surveyor^®^ Mutation Detection Kit; Cat#706025, IDTdna.com) or the Guide-it Resolvase (Guide-it™ Mutation Detection Kit, Cat#631443, Clontech) according to the recommended instructions. We also explored if a gene, when transiently expressed in *N. benthamiana* can also be mutated. For this, a plant expression binary plasmid, pGWB415-HA-YFP, carrying a 35S promoter driving a HA-YFP fusion gene was co-transformed with either pDe-Cas9-D10A-gYFP or pDe-Cas9-D10A-gNbPDS (as negative control) into wild type *N. bethamiana* leaves and assayed for mutation in the *YFP* gene using mismatch-specific nuclease assays as above.

## Additional file

**Additional file 1.** This file contains two Figures as follows. **Figure S1.** Schematics illustrating sequences and positions of primers relative to the final CRISPR RNA. **Figure S2**. Restriction fragment length polymorphism (RFLP) analysis of YFP CRISPR RNA.

## Abbreviations

gRNA: guide RNA (protospacer + crRNA + tracrRNA)
sgRNA: single guide RNA (crRNA + tracrRNA)
CRISPR: clustered, regulatory interspaced, short palindromic repeats
Cas9: CRISPR-associated 9
PAM: protospacer-adjacent motif
crRNA: CRISPR RNA
tracrRNA: trans-activating crRNA
