# Supplementary material for: A highly efficient ligation-independent cloning system for CRISPR/Cas9 based genome editing in plants
Source: Plant Methods. 2017 Oct 16;13:86. doi: 10.1186/s13007-017-0236-9 (PMC5644101; doi:10.1186/s13007-017-0236-9)
Supplement: Supplementary file 1 — Additional file 1. This file contains two Figures as follows. Figure S1. Schematics illustrating sequences and positions of primers relative to the final CRISPR RNA. Figure S2. Restriction fragment length polymorphism (RFLP) analysis of YFP CRISPR RNA. [file 13007_2017_236_MOESM1_ESM.docx]

**A highly efficient ligation-independent cloning system for CRISPR/Cas9 based genome editing in plants**

Aftab A. Khan^#^, Ashraf El-Sayed ^#^, Arianna Mangravita–Novo^#^, Shaheen Bibi, Zunaira Afzal, David J. Norman and Gul Shad Ali^*^

Mid-Florida Research and Education Center, and Department of Plant Pathology, University of Florida, Institute of Food and Agriculture Sciences, 2725 S. Binion Road, Apopka, FL 32703.

Aftab A. Khan (aftabkhan@ufl.edu)

Ashraf El-Sayed (ashrafsabry@zu.edu.eg)

Arianna Mangravita–Novo (amangra@ufl.edu)

Shaheen Bibi (shaheen110@ufl.edu)

Zunaira Afzal (zunairaafzal@ufl.edu)

David J. Norman (djn@ufl.edu)

^*^ Corresponding author: Gul S. Ali, E-mail address: gsali@ufl.edu

^#^ These authors contributed equally to this work.

**Additional information**

**
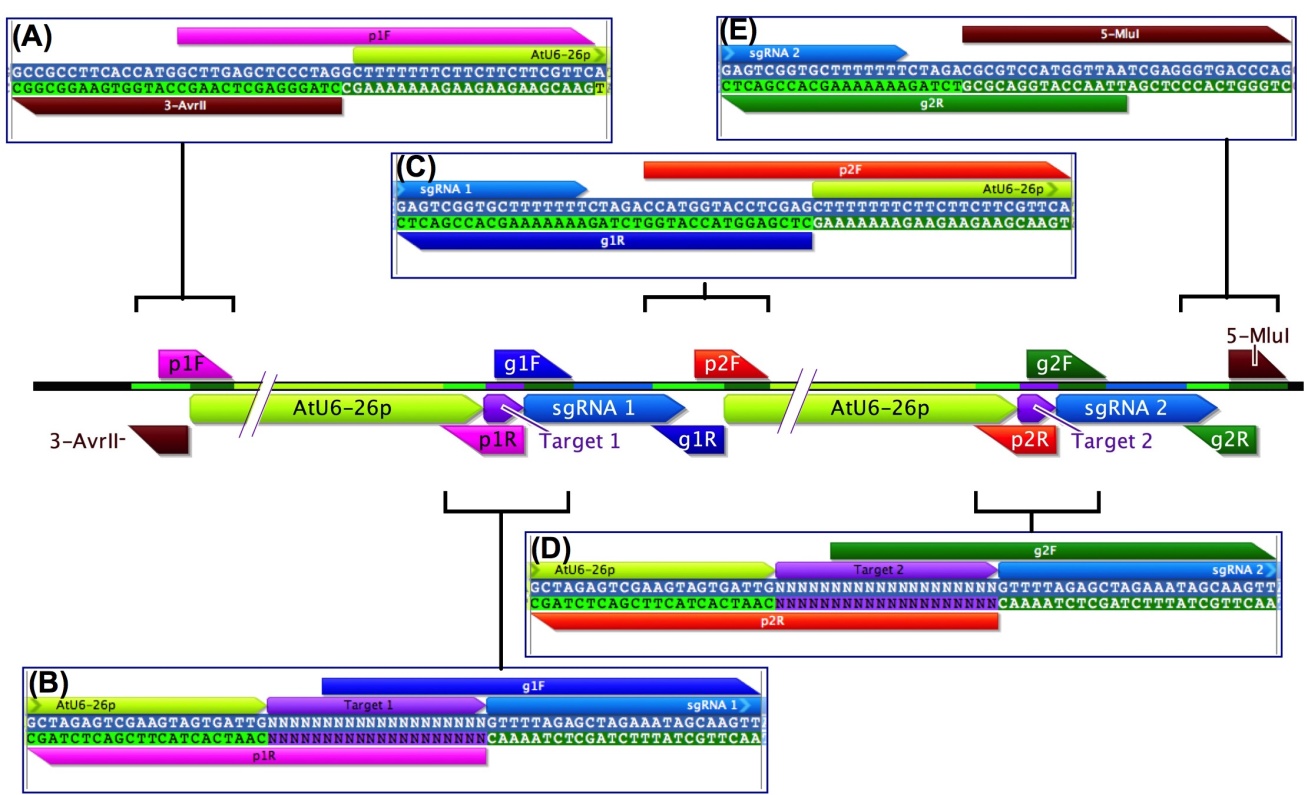
**

**Figure S1. Schematics illustrating sequences and positions of primers relative to the final CRISPR RNA**

**(A)** Primer p1F (marked by magenta forward arrow) is a forward primer, which consists of a 20-nucleotide 3` region spanning the 5` end of the AtU6-26 promoter (marked by green bar), and a 15-bp sequence at its 5` end, which overlaps with a 15-bp sequence located at the 5` end of the reverse primer, 3-AvrII (marked by brown arrow). The 3` end of primer 3-AvrII corresponds to sequence located at the 3` end of the backbone of vector pDe-Cas9 or pUC57GW. **(B)** Primer p1R (marked by magenta reverse arrow), which consists of a 22-bp 3` region which is complementary to the 3` end of AtU6-26 promoter, and a 20 nucleotide protospacer for sgRNA 1 (denoted by 20N). Primer g1F (marked by blue forward arrow) consists of a 3` 25-bp region spanning the 5` end of sgRNA (marked by sgRNA1), and a 5` 15-bp sequence, which overlaps with the 5` 15N of p1R primers. **(C)** Primer g1R (marked by blue reverse arrow) consists of a 3` 17-bp region, which is complementary to the 3’ end of sgRNA, and a 20-bp 5` region, the 1^st^ 15 bp region of which overlaps with the 5`15 bp region of primer p2F (marked by red forward arrow). The last 22 bp of p2F corresponds to the 5` end of AtU6-26 promoter. **(D)** Primer p2R (marked by red reverse arrow), which consists of a 22-bp 3` region which is complementary to the 3` end of AtU6-26 promoter, and a 20 nucleotide protospacer for sgRNA 2 (denoted by 20N). Primer g2F (marked by green forward arrow) consists of a 3` 25-bp region spanning the 5` end of sgRNA (marked by sgRNA2), and a 5` 15-bp sequence, which overlaps with 5` 15N of p2R primers. **(E)** Primer g2R (marked by green reverse arrow) consists of a 3` 17-bp region, which is complementary to the 3’ end of sgRNA (marked by sgRNA2), and a 20-bp 5` region, the 1^st^ 15 bp region of which overlaps with the 5`15-bp region of primer 5-MluI (marked by brown forward arrow). The 3` end of primer 5-MluI corresponds to sequence located at the 5` end of the backbones of vector pDe-Cas9 or pUC57GW.

**
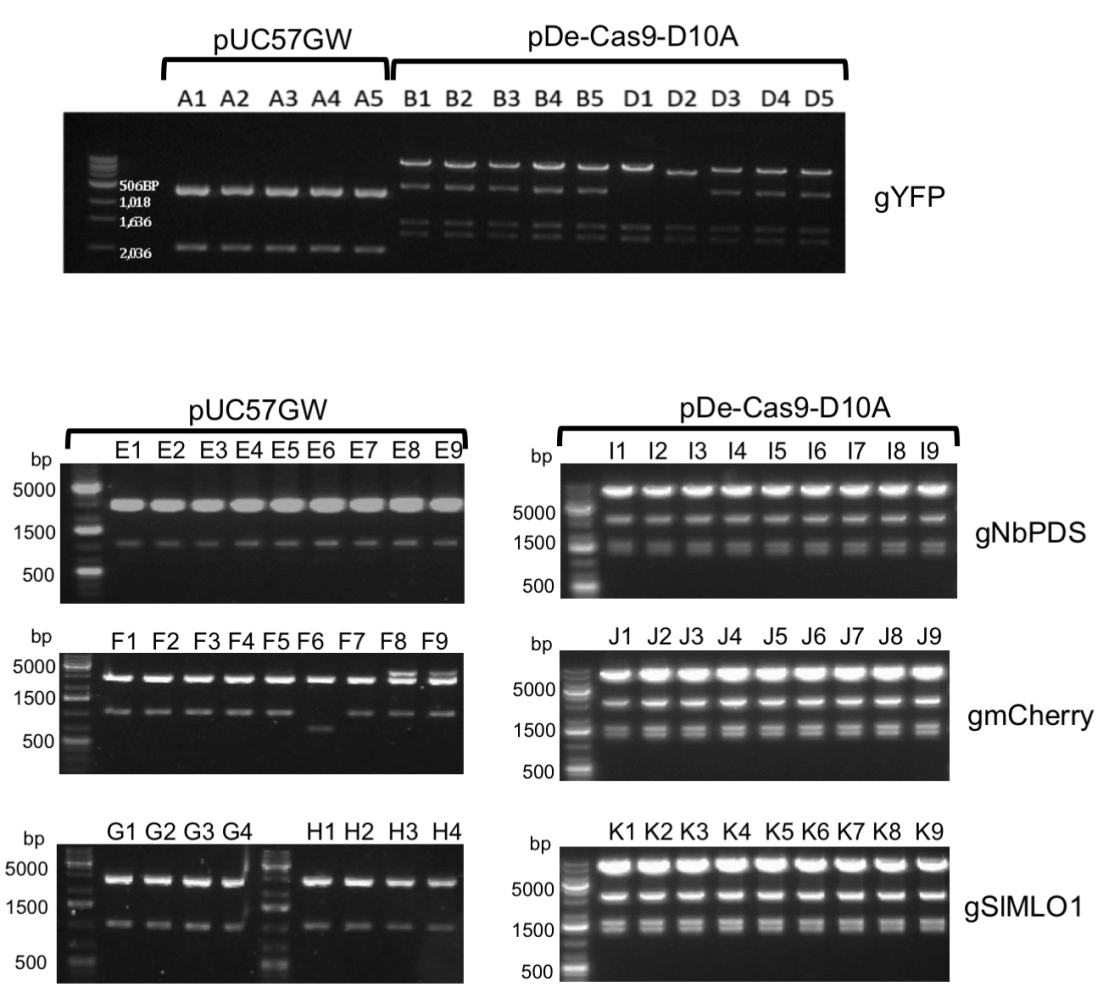
**

**Figure S2. Restriction fragment length polymorphism (RFLP) analysis of YFP CRISPR RNA**

RFLP analysis of plasmid isolated from random colonies of clones in pUC57GW of YFP-gRNA1 and YFP-gRNA2 (A1-A5) and pDe-Cas9-D10A (B1-B5) using strategy that uses two rounds of PCR. D1-D5 shows RFLP analysis using strategy that uses one round of PCR by all four fragments A, B, C and D after Round 1 PCR with pDe-Cas9-D10A. Similar results with one round of PCR were obtained for making gNbPDS (E1 – E9), gmCherry (F1 – F9) and gSlMLO1 (G1 – G4, H1 – H4) constructs in pUC57GW, and and gNbPDS (I1 – I9), gmCherry (J1 – J9) and gSlMLO1 (K1 – K9) in pDe-Cas9-D10A.
